# Automated cervical cell segmentation using deep ensemble learning

**DOI:** 10.1186/s12880-023-01096-1

**Published:** 2023-09-21

**Authors:** Jie Ji, Weifeng Zhang, Yuejiao Dong, Ruilin Lin, Yiqun Geng, Liangli Hong

**Affiliations:** 1 Network & Information Center, Shantou University, Shantou, 515041 Guangdong China; 2 Guangdong Provincial International Collaborative Center of Molecular Medicine, Laboratory of Molecular Pathology, Shantou University Medical College, Shantou, 515041 China; 3 Department of Pathology, the First Affiliated Hospital of Shantou University Medical College, Shantou, 515041 Guangdong China

**Keywords:** Cervical cell segmentation, Cervical cytology screening, U-Net, U-Net +  +, Deep ensemble learning

## Abstract

**Background:**

Cervical cell segmentation is a fundamental step in automated cervical cancer cytology screening. The aim of this study was to develop and evaluate a deep ensemble model for cervical cell segmentation including both cytoplasm and nucleus segmentation.

**Methods:**

The Cx22 dataset was used to develop the automated cervical cell segmentation algorithm. The U-Net, U-Net +  + , DeepLabV3, DeepLabV3Plus, Transunet, and Segformer were used as candidate model architectures, and each of the first four architectures adopted two different encoders choosing from resnet34, resnet50 and denseNet121. Models were trained under two settings: trained from scratch, encoders initialized from ImageNet pre-trained models and then all layers were fine-tuned. For every segmentation task, four models were chosen as base models, and Unweighted average was adopted as the model ensemble method.

**Results:**

U-Net and U-Net +  + with resnet34 and denseNet121 encoders trained using transfer learning consistently performed better than other models, so they were chosen as base models. The ensemble model obtained the Dice similarity coefficient, sensitivity, specificity of 0.9535 (95% CI:0.9534–0.9536), 0.9621 (0.9619–0.9622),0.9835 (0.9834–0.9836) and 0.7863 (0.7851–0.7876), 0.9581 (0.9573–0.959), 0.9961 (0.9961–0.9962) on cytoplasm segmentation and nucleus segmentation, respectively. The Dice, sensitivity, specificity of baseline models for cytoplasm segmentation and nucleus segmentation were 0.948, 0.954, 0.9823 and 0.750, 0.713, 0.9988, respectively. Except for the specificity of cytoplasm segmentation, all metrics outperformed the best baseline models (*P* < 0.05) with a moderate margin.

**Conclusions:**

The proposed algorithm achieved better performances on cervical cell segmentation than baseline models. It can be potentially used in automated cervical cancer cytology screening system.

**Supplementary Information:**

The online version contains supplementary material available at 10.1186/s12880-023-01096-1.

## Background

Cervical cancer is a common malignancy that poses a serious threat to women’s health. It is the fourth most common cancer in terms of both incidence and mortality. In 2020, approximately 600,000 new cases of cervical cancer were diagnosed and more than 340,000 people died from this disease globally [1, 2]. Fortunately, cervical cancer has a long precancerous stage, and annual screening programs can help detect and treat it in a timely manner. If cervical cancer is detected early, it can be completely eradicated. At present, manual screening of abnormal cells from a cervical cytology slide is still the common practice. However, it is usually tedious, inefficient and high-cost. Consequently, the automated cervical cancer cytology screening has attracted increasing attention. In the past few years, deep learning (DL), a branch of machine learning, has made great success in the field of medical image analysis [3–5]. The segmentation of cervical cytology images plays an important role in the automated cervical cancer cytology screening [6]. However, the performance of cervical cell segmentation is far from perfect [6–10].

Different from histology, which involves examining an entire section of tissue, cytology generally focuses on individual cells or clusters of cells. In some cases, several cells can determine the diagnostic result of the whole slide. One of the mainstream methods for automated cervical cancer cytology screening is cell segmentation followed by single cell classification. Compared to cervical cell segmentation, more research has been conducted on cell classification and more public datasets have been released [11–14]. According to the 2014 Bethesda guideline [15], nuclear morphologies, which include nuclear size and shape, nuclear pleomorphism, nucleus-to-cytoplasm ratio, multiple nuclei, and nucleoli morphology, are the most important biomarkers in cervical cytology screening. Therefore, both cytoplasm segmentation and nucleus segmentation are important for automated cervical cytology screening.

Previous studies have some limitations. Some previous studies only segmented cytoplasm or nucleus (not both of them simultaneously) [16]. Moreover, a lot of research was based on very limited data, so the generalization ability of these algorithms is not guaranteed. For example, some research only used 8 real cervical cytology images and over a hundred synthetic images [9, 10]. To the best of our knowledge, all previous studies adopted a single CNN such as the standard U-Net and did not use transfer learning during training [6]. Deep learning system heavily relies on the amount and quality of data. So far, there exist some public cervical cell segmentation datasets including ISBI2014 [9], ISBI2015 [10], BTTFA [16] and Cx22 dataset [6]. Among them the recently released Cx22 dataset is the biggest publicly available cervical cell segmentation dataset and contains both cytoplasm and nuclei annotations. The data descriptor paper of the Cx22 dataset also provided multiple baseline models including U-Net [17], U-Net +  +  [18] and U-Net +  +  +  [19], however performances of these baseline models are far from perfect. The Dice, sensitivity, specificity for cytoplasm segmentation and nucleus segmentation were 0.948, 0.954, 0.9823 and 0.750, 0.713, 0.9988, respectively.

This study aimed to develop a automated cervical cell segmentation algorithm including both cytoplasm and nucleus segmentation By means of a relatively large dataset, different model architectures with different encoders, model ensemble and loading pre-trained encoder weights, our algorithm outperformed those of previous studies.

## Methods

### Dataset and data processing

The Cx22 dataset delineate the contours of 14,946 cellular instances in 1320 images that were generated by a label cropping algorithm based on the region of interest. The data source and annotation pipeline were described in detail in the data descriptor paper [6]. A representative image and its ground truth labels can be found in the results section. The Cx22 dataset stored data using MATLAB.mat files with hdf5 data format. For convenience, these files were converted into image and mask files with jpeg format using Python code. The Cx22 dataset contained a training dataset and a testing dataset with 400 and 100 samples, respectively. Every sample consists of an image and two mask files, one for cytoplasm annotation and the other for nuclei annotation. All images have a resolution of 512*512 pixels. For model selection and hyperparameter tuning, the training dataset was further split into a new training dataset and a tuning dataset with a ratio of 0.9 and 0.1. The Cx22 dataset contains a predefined test dataset and the sample size of test dataset is not very small, for the convenience of comparing the performance our algorithm with that of baseline, in this study cross validation was not adopted.

### Overall architecture

In this study, both cytoplasm segmentation and nucleus segmentation were considered as semantic segmentation tasks. These two tasks can be solved by either one multi-class classifier or two independent binary-class classifiers. To decouple the interference between cytoplasm segmentation and nucleus segmentation and simplify the hyper-parameter setting process, the latter method was adopted. According to common practice, the positive class stands for cytoplasm or nucleus and the negative class for background.

**Fig. 1:**
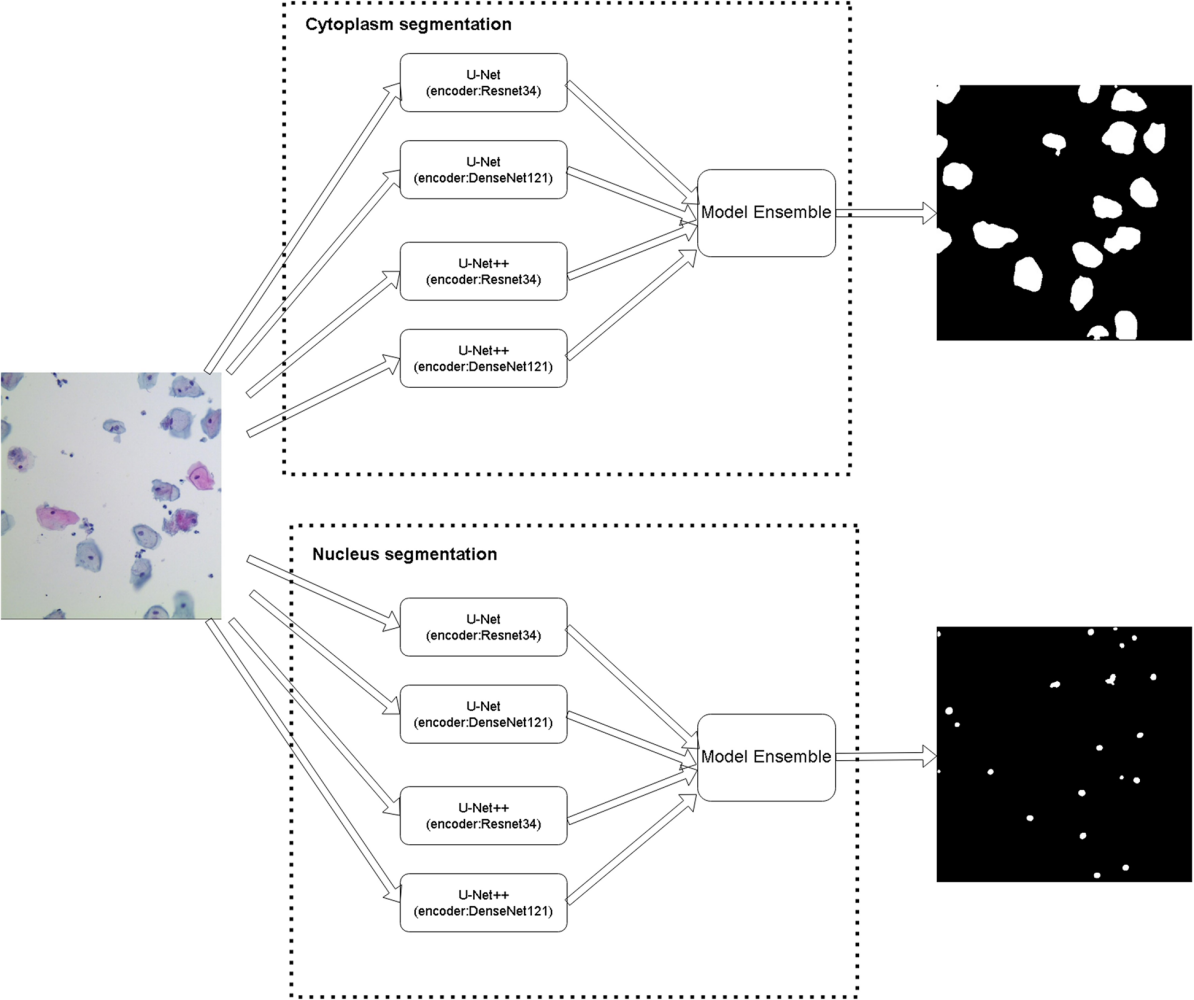
The flowchart of automated cervical cell segmentation. The two dashed boxes demonstrate two ensemble models, one for cytoplasm segmentation and the other for nucleus segmentation. The model ensemble method is unweighted average

The flowchart of the automated cervical cell segmentation algorithm is shown in Fig. 1. Given an image, cytoplasm and nucleus were segmented independently. For every segmentation task, the image was inputted to multiple base models. The final predictions were obtained by aggregating results from multiple models using model ensemble method.

### Base models

To get a good ensemble model, base models should be as more accurate as possible, and as more diverse as possible [20]. Six different model architectures specifically U-Net, U-Net +  + , DeepLabV3 [21] DeepLabV3Plus [22], Transunet [23], and Segformer [24] were chosen as candidate models. These models belong to three different architectures, i.e., encoder-decoder, dilated convolution and vision transformer, and all of which were widely used. Some other U-Net variants including attention U-Net [25], R2U-Net [26] were also tested during pre-experiments on this tasks, because they did not perform better than U-Net and U-Net +  + and consume more GPU memory, they were abandoned in this study. Likewise, Swin transformer for semantic segmentation model [27] was not adopted because during pre-experiments on other tasks it did not perform better than its counterpart Transunet and Segformer models.

For every U-Net and U-Net +  + model, two different encoders resnet34 and densenet121 were used. Likewise, resnet34 and resnet50 were used as encoders of every DeepLabV3 and DeepLabV3Plus model. Densenet121 was replaced by resnet50 in DeepLabV3-series models was because there exist some bugs related to DeepLabV3-series models in the SMP implementation [16]. For the architecture of TransUnet and SegFormer, only the default setting was used. Setting of Transunet: vit_blocks = 12, vit_heads = 12, vit_dim_linear_mhsa_block = 3072, patch_size = 8, vit_transformer_dim = 768, vit_transformer = None, vit_channels = None. Setting of SegFormer: dims = (32, 64, 160, 256), heads = (1, 2, 5, 8), ff_expansion = (8, 8, 4, 4), reduction_ratio = (8, 4, 2, 1), num_layers = 2, decoder_dim = 256. Model implementation details can be found in the source code. For convenience, if a model has both an architecture name and an encoder name, it was named by combining the architecture name and encoder name. For example, Unet_resnet34 means that the model has the U-Net architecture and resnet34 encoder. Characteristics of candidate base models are shown in Table 1.

**Table 1:** Characteristics of candidate base models

Model	Parameters	Encoder	Decoder
Unet_resnet34	24,436,369	33 conv layers	10 conv layers
Unet_densenet121	13,607,633	117 conv layers	10 conv layers
UnetPlusPlus_resnet34	26,078,609	33 conv layers	10 conv layers
UnetPlusPlus_densenet121	30,072,273	117 conv layers	10 conv layers
DeepLabV3_resnet34	26,007,105	33 conv layers	1 conv layer + ASPP
DeepLabV3_resnet50	39,633,729	49 conv layers	1 conv layer + ASPP
DeepLabV3Plus_resnet34	22,437,457	33 conv layers	2conv layers + ASPP
DeepLabV3Plus_resnet50	26,677,585	49 conv layers	2conv layers + ASPP
Segformer	7,717,473	/	/
Transunet	67,875,963	/	/

Conv layer and ASPP stand for convolutional layer and atrous spatial pyramid pooling layer, respectively. Segformer and Transunet are transformer-based models, their encoder and decoder structures are not listed

These models were trained independently, afterwards model selection was conducted based on performance metrics. Finally, four models, i.e., Unet_resnet34, Unet_densenet121, UnetPlusPlus_resnet34 and UnetPlusPlus_densenet121 were chosen as the base models. Model performance comparisons were depicted in the results section.

### Ensemble model

Although the performance differences among all models were significant, the performance differences among selected models were very small. Multiple ensemble methods, which include weighted averaging (using validation loss as weighting factor), unweighted averaging and stacking, were tested in preliminary experiments. Even though any ensemble method performed better than any single model, there was no obvious difference in the performance of the using different ensemble methods. For simplicity, unweighted average was chosen as the model ensemble method [20, 28]. It not only eliminated the need of setting parameters in weighted average or training a new model in stacking, but also did not decrease performance. Given an image, for each pixel four base models independently gave their predicted probabilities. The number of base models was set to 4 was because further increasing the number of base models would not result in perceivable performance improvement, but it would increase training time and slow down inference speed. The final probabilities were obtained by aggregating these outputted probabilities of multiple models using the unweighted average method. If its predicted probability was above a predefined threshold, the pixel was considered as positive, otherwise negative. For simplicity, the default value of 0.5 was used as the cut-off value. The mathematical formula for every pixel prediction is:

$$\mathrm{pred}\_\mathrm{class}=\frac{\sum_{i=1}^M\;p_i}M>0.5$$

For a pixel, p_i_ is the predicted probability of model No i. M is the number of base models and in this case is equal to 4. If pred_class is true, the pixel is predicted as cytoplasm or nucleus depending on the segmentation task.

### Training strategies

The sample size of Cx22 is not large, so real-time image augmentation was adopted during training to avoid overfitting. Compared with beforehand image augmentation, real-time image augmentation is more flexible. Image augmentation included random horizontal and vertical flipping, random brightness and contrast modifications, gaussianBlur transformation, hue/saturation color transformation and among others were used. Image augmentation was implemented with the albumentations library and PyTorch dataset class.

The data distribution of cytoplasm segmentation was relatively balanced, so binary cross-entropy was used as the loss function of cytoplasm segmentation. However, the nucleus occupies only a small area of the image, to tackle this class imbalance weighted binary cross-entropy was used as the loss function of cytoplasm segmentation and the weight factor for positive class was set to 8. Compare with similarity based loss functions such as the Dice loss and IOU loss, the binary cross-entropy loss has smooth gradients [29] and so as to train faster.

For models except for SegFormer and Transunet, encoders have corresponding easy to obtain ImageNet pre-trained models. Consequently, these models were trained under two settings: trained from scratch, encoders initialized from ImageNet pre-trained models and then all layers were fine-tuned.

Adam [30] with lookahead [31] (k = 5, alpha = 0.5) was used as the optimizer. Automatic mixed precision training [32] was used to speed up the training and inference processes and save GPU memory. Label smoothing (ε = 0.1) was used to calibrate probabilities and improve generalizability [33]. The batch size was set to 32 and the number of epochs were set to 20. The initial learning rate was set to 1e-3, and multiplied by a factor of 0.1 at 30%, 60% and 90% of the training epochs. Every model was trained 3 times under the same setting, and the model with the minimum validation loss was chosen as the final model. During training, performances were not sensitive to these hyper-parameters.

### Evaluation metrics

In the original Cx22 data descriptor paper, the Dice, true positive rate (sensitivity) and false positive rate (1-specificity) [34] were used to quantitatively assess baseline models. To make a fair comparison, in this study these same performance metrics were used.

$$\mathrm{Sensitivity}=\frac{TP}{TP+FN}$$

$$\mathrm{Specificity}=\frac{TN}{TN+FP}$$

$$\mathrm{Dice}=\frac{2TP}{2TP+TN+FP}$$

A *P* value of less than 0.05 was considered statistically significant. Bootstrap method on the pixel level with a resampling number of 500 was used to calculate the 95% CIs. For simplicity, confidence intervals only calculated on performance indicators of ensemble models.

## Experimental settings

Hardware: Intel Core i7-10,700, 128 GB Memory, Nvidia GTX 3090 * 2.

Software: Ubuntu 20.04, Cuda 11.3, Anaconda 4.10.

Programming language and libraries: Python 3.8, Pytorch 1.10, Torchvision OpenCV, NumPY, SciPY, Sklearn, Matplotlib, Pandas, Albumentations, segmentation_models_pytorch, Tqdm. Detailed information about these software libraries can be found in the file requirements.txt of the source code.

## Results

Training and validation loss curves were used to demonstrate convergence speed and determine whether there exists overfitting. Loss curve graphs of cytoplasm segmentation and of nucleus segmentation are shown in the supplement Figure S1 and Figure S2, respectively. These graphs illustrate that the training speed of these models is fast and there is no obvious overfitting. The reason loss curves of Transunet Segformer models were not included is that during training some models did not converge and performances of other models were pretty bad.

All performance analyses were conducted on the testing dataset. Performance comparison of different models trained from scratch is shown in Table 2.

**Table 2:** Performance comparison of base models trained from scratch

Task Type	Model	Dice	Sensitivity	Specificity
Cytoplasm	Unet_resnet34	0.926	0.9275	0.9777
	Unet_densenet121	0.9259	0.9202	**0.9801**
	UnetPlusPlus_resnet34	0.9229	0.926	0.9762
	UnetPlusPlus_densenet121	**0.9289**	**0.953**	0.9708
	DeepLabV3_resnet34	0.9146	0.918	0.9736
	DeepLabV3_resnet50	0.9159	0.9393	0.967
	DeepLabV3Plus_resnet34	0.9282	0.948	0.9721
	DeepLabV3Plus_resnet50	0.924	0.9323	0.9747
	Transunet	/	/	/
	Segformer	0.8717	0.8894	0.9554
Nucleus	Unet_resnet34	0.6299	0.8504	0.9931
	Unet_densenet121	0.6676	0.9017	0.9935
	UnetPlusPlus_resnet34	0.6966	0.9212	**0.9941**
	UnetPlusPlus_densenet121	**0.697**	**0.9287**	**0.9941**
	DeepLabV3_resnet34	0.5326	0.8715	0.9887
	DeepLabV3_resnet50	0.531	0.8917	0.9881
	DeepLabV3Plus_resnet34	0.5237	0.8127	0.9896
	DeepLabV3Plus_resnet50	0.5593	0.8129	0.9912
	Transunet	/	/	/
	Segformer	/	/	/

In the first column, cytoplasm and nucleus stand for the cytoplasm segmentation task and the nucleus segmentation task, respectively. The symbol “/” indicates that the model is collapsed as it predicts all pixels as negative or positive. Bold values represent the best results

Performance comparison of different models, which encoders were initialized by corresponding ImageNet pre-trained models, is shown in Table 3.

**Table 3:** Performance comparison of base models, which encoders were initialized from ImageNet pre-trained models

Task Type	Model	Dice	Sensitivity	Specificity
Cytoplasm	Unet_resnet34	0.9497	0.9596	0.9819
	Unet_densenet121	0.9527	**0.9625**	0.9828
	UnetPlusPlus_resnet34	**0.9533**	0.9616	0.9835
	UnetPlusPlus_densenet121	0.9525	0.9594	**0.9837**
	DeepLabV3_resnet34	0.9407	0.9496	0.9795
	DeepLabV3_resnet50	0.9386	0.9492	0.9783
	DeepLabV3Plus_resnet34	0.9455	0.9475	0.9833
	DeepLabV3Plus_resnet50	0.9494	0.9598	0.9817
Nucleus	Unet_resnet34	0.7411	0.9431	0.9951
	Unet_densenet121	0.7506	0.9566	0.9952
	UnetPlusPlus_resnet34	**0.8055**	0.9481	**0.9967**
	UnetPlusPlus_densenet121	0.7731	**0.9653**	0.9957
	DeepLabV3_resnet34	0.6088	0.947	0.9906
	DeepLabV3_resnet50	0.6419	0.9506	0.9918
	DeepLabV3Plus_resnet34	0.6721	0.9053	0.9936
	DeepLabV3Plus_resnet50	0.7353	0.9483	0.9949

In the first column, cytoplasm and nucleus stand for the cytoplasm segmentation task and the nucleus segmentation task, respectively. Bold values represent the best results

As shown in Table 2, in all cases, the U-Net-series models were consistently better than the DeeplabV3-series models. No matter on which segmentation task and what the model architecture was used, compared with training from scratch, using the ImageNet pretrained encoders apparently improved the performances. Even though Transunet [23] and Segformer [24] obtained very good or even SOTA results on many image segmentation benchmarks, in this study they performed much worse than their CNN counterparts. In most cases, these models even collapsed and predicted all pixels as negative or positive. Finally, according to performance metrics, for every segmentation task, 4 models Unet_resnet34, Unet_densenet121, UnetPlusPlus_resnet34, and UnetPlusPlus_densenet121 were chosen as base models, all of which were trained by the transfer learning strategy.

**Fig. 2:**
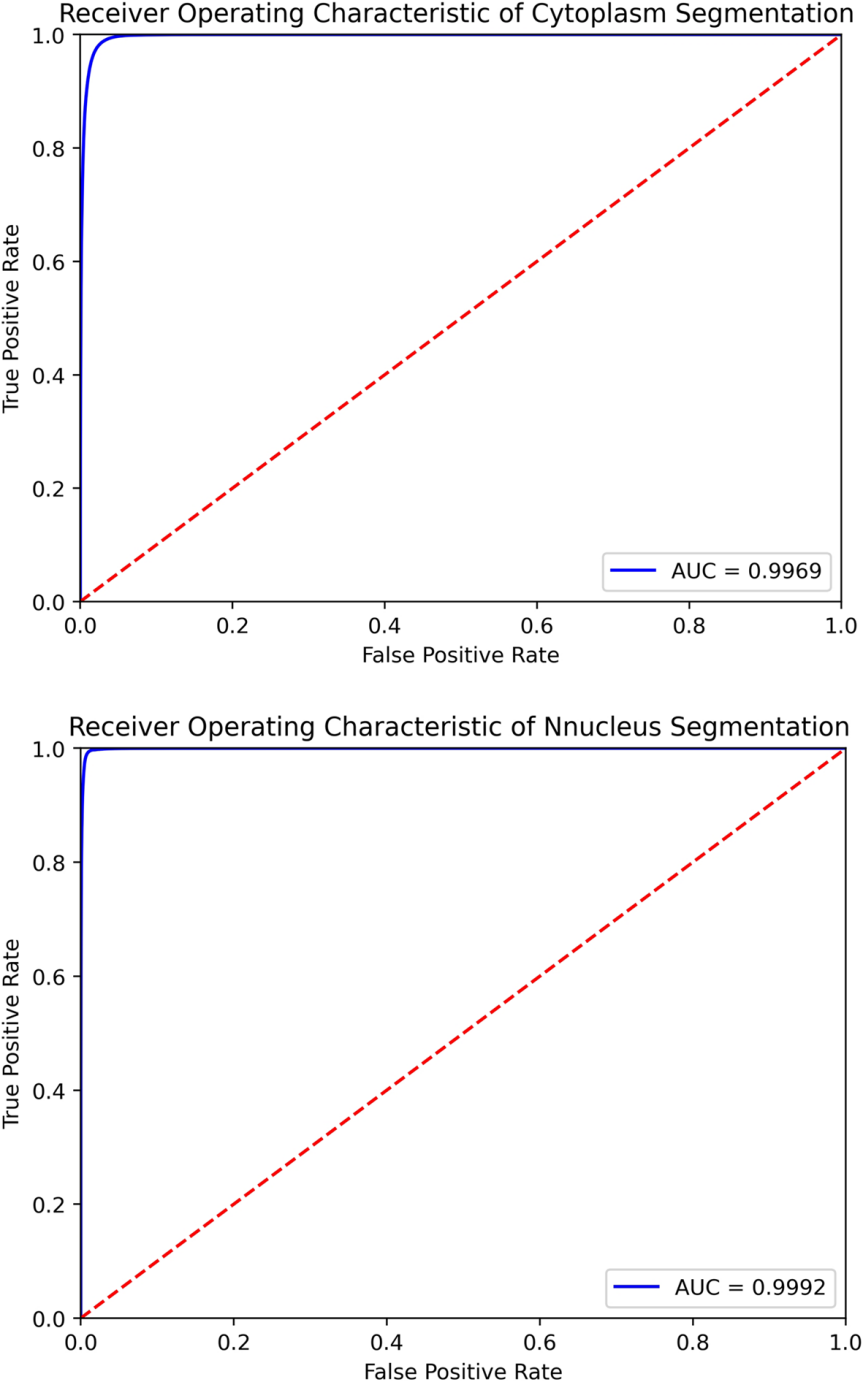
The ROC curves including AUC scores of cytoplasm segmentation and nucleus segmentation

Although not every performance indicator of the ensemble model was better than that of any single model, all performance metrics of the ensemble model were better than the arithmetic mean of performance metrics of base models. Performance comparison of ensemble models and the arithmetic means of base models on the testing dataset is depicted in Table 4. The performance metrics of ensemble models were better than arithmetic means of performance metrics of base models (*P* < 0.05). ROC curves including AUC scores of cytoplasm segmentation and nucleus segmentation are shown in Fig. 2.

**Table 4:** Performance comparison of ensemble models and the arithmetic means of base models on the testing dataset

Task Type	Model	Dice	Sensitivity	Specificity
Cytoplasm	Ensemble model	**0.9535** (0.9534–0.9536)	**0.9621** (0.9619–0.9622)	**0.9835** (0.9834–0.9836)
	Average value	0.9521	0.9601	0.9830
Nucleus	Ensemble model	**0.7863** (0.7851–0.7876)	**0.9581** (0.9573–0.959)	**0.9961** (0.9961–0.9962)
	Average value	0.7676	0.9533	0.9957

In the first column, cytoplasm and nucleus stand for the cytoplasm segmentation task and the nucleus segmentation task, respectively. For every task, the first row depicts performance metrics of the ensemble model and the second row depicts the average performance metrics of base models. Bold values represent the best results, and confidence intervals are depicted in brackets

The data descriptor paper [6] also provided multiple baseline models including U-Net, U-Net +  + and U-Net +  +  + . In this study, for every task we chose the best baseline metrics to compare. Performance comparison of the baseline model and the ensemble model is shown in Table 5. Except for the specificity on nucleus segmentation, the ensemble model outperformed the best baseline model with a moderate margin on all tasks. The specificity on nucleus segmentation of the ensemble model was very close to that of baseline model, and both were near perfect.

**Table 5:** Performance comparison of baseline and ensemble models on the testing dataset

Task Type	Model	Dice	Sensitivity	Specificity
Cytoplasm	Best Baseline Model	0.948	0.954	0.9823
	Ensemble model	**0.9535** (0.9534–0.9536)	**0.9621** (0.9619–0.9622)	**0.9835** (0.9834–0.9836)
Nucleus	Best Baseline Model	0.750	0.713	**0.9988**
	Ensemble model	**0.7863** (0.7851–0.7876)	**0.9581** (0.9573–0.959)	**0.9961** (0.9961–0.9962)

In the first column, cytoplasm and nucleus stand for the cytoplasm segmentation task and the nucleus segmentation task, respectively. Bold values represent the best results, and confidence intervals are depicted in brackets

**Fig. 3:**
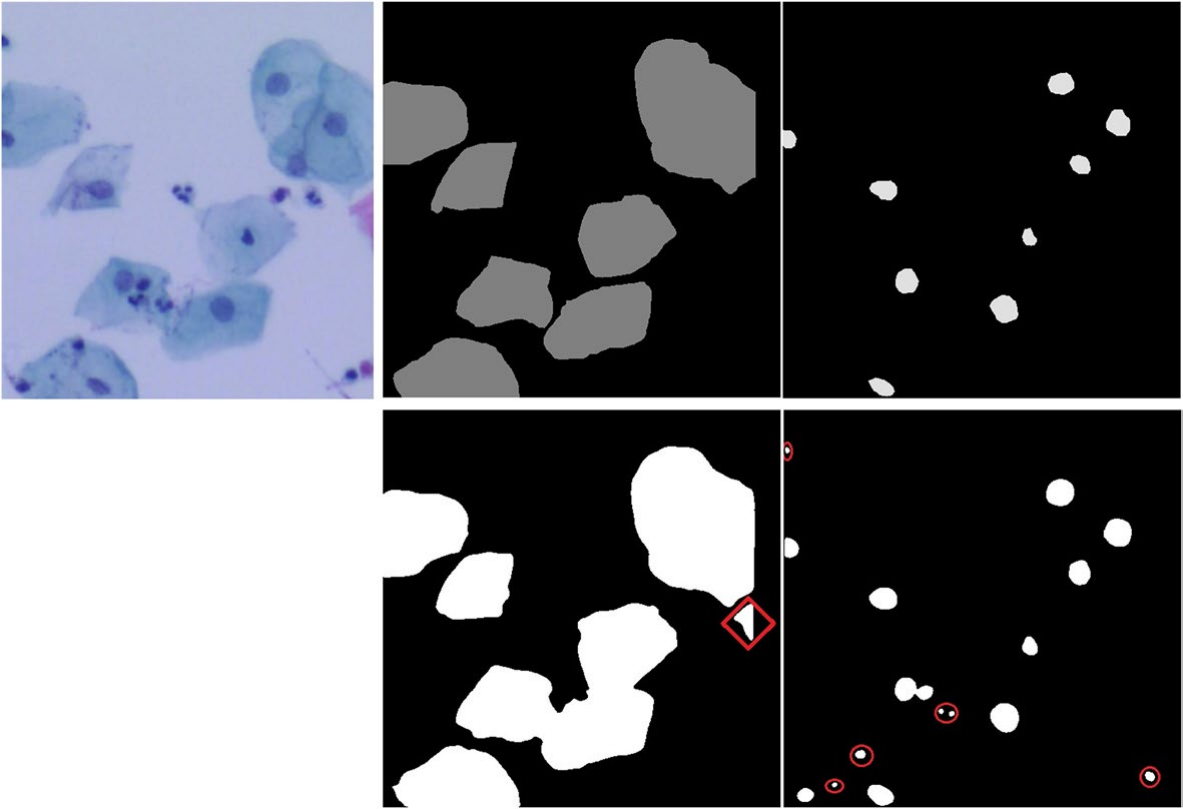
A representative image, its ground truth annotations and predicted masks. The image, ground truth annotations are shown in the first row. Predicted masks are shown in the second row. Cytoplasm images and nucleus images are shown in the second and third column, respectively

Besides quantitative analyses, qualitative analyses were also conducted in this study. From a human's subjective point of view, predicted masks were very close to ground truth annotations. A randomly selected case including the image, its ground truth annotations and predicted masks are shown in Fig. 3. It should be mentioned that most of these false positives are not actually false positives. The region marked by red color in the predicted cytoplasm image is a cytoplasm area. Because the main part of the cell was cropped by its neighbor image, the remaining small portion of cytoplasm was not labeled. Likewise, the noise areas in the predicted nucleus image marked by red circles are small nucleus neglected by human annotations.

## Discussion

Based on the above results, the following assumptions were proposed: under the conditions of medical image segmentation with small to medium sample size, U-Net variants are better than DeeplabV3 variants, and vision transformer models are much worse than CNNs. Vision transformers have fewer priors so that they need more training data. Even though both Transunet and Segformer adopt a CNN-like hierarchical structure and using a few convolutional layers at the lower level, they still need more data to train than U-Net variants. Whether these assumptions hold true for medical image segmentation tasks other than cervical cytology cell segmentation should be further investigated.

This study has both strengths and limitations. The strengths of this study include on the cytoplasm segmentation task, the proposed ensemble model outperformed the best baseline model on all performance metrics with a moderate margin. And on the nucleus segmentation task, the proposed ensemble model outperformed the best baseline model on all performance metrics except for specificity with a moderate margin. Moreover, this study compared the performances of different model architectures, different encoders, and different training strategies. These comparison results may be extended to other medical image segmentation tasks. This study also has some limitations. First and most importantly, cells are important objects in cervical cancer cytology screening, and both cytoplasm and nuclei are important parts of a cell. However, the semantic segmentation models only classify every pixel, they do not identify objects. Regarding to this issue, both adding a post-processing algorithm after the semantic segmentation model to do object identification and using instance segmentation algorithm are feasible solutions. Unfortunately, both solutions will bring a certain degree of complexity. Second, this study only used the Cx22 dataset, the generalization ability of the models was not guaranteed. We plan to conduct a new study in the future, which will add the ability of cell object identification and carry out external validation.

## Conclusions

In this study, we have developed an automated cervical cytology cell segmentation algorithm using the Cx22 dataset by means of deep ensemble learning. The algorithm obtained the Dice, sensitivity, and specificity of 0.9535 (CIs:0.9534–0.9536), 0.9621 (0.9619–0.9622), 0.9835 (0.9834–0.9836) and 0.7863 (0.7851–0.7876), 0.9581 (0.9573–0.959), 0.9961 (0.9961–0.9962) for cytoplasm segmentation and nucleus segmentation, respectively. On most performance metrics, our algorithm outperformed the best baseline models (*P* < 0.05) with a moderate margin. In the future, after adding the cell identification functionality and conducted sufficient external validation, it can be used in automatic cervical cancer cytology screening system.

### Supplementary Information

**Additional file 1: Figure S1** and **Figure S2.**

## Data Availability

The used datasets were obtained from publicly open-source datasets from https://github.com/LGQ330/Cx22. The source code and trained models are publicly available at https://github.com/linchundan88/cervical_cell_sem_seg and http://wechat.stu.edu.cn/cervical_cell_seg_models/trained_models.zip, respectively.

## Abbreviations

CNN: Convolutional neural network
ASPP: Atrous spatial pyramid pooling layer;
DICE: Dice similarity coefficient
FP: False positive
FN: False negative
TP: True positive
TN: True negative
ROC: Receiver operating characteristic
AUC: Area under the receiver operating characteristic curve
CL: Confidence interval
SOTA: State-of-the-art
SMP: Segmentation model pytorch

## References

[1] Sung H, Ferlay J, Siegel RL, Laversanne M, Soerjomataram I, Jemal A (2021). Global cancer statistics 2020: GLOBOCAN estimates of incidence and mortality worldwide for 36 cancers in 185 countries. CA Cancer J Clin..

[2] Siegel RL, Miller KD, Jemal A (2020). Cancer statistics, 2020. CACancer J Clin..

[3] Cen L-P, Ji J, Lin J-W, Ju S-T, Lin H-J, Li T-P, et al. Automatic detection of 39 fundus diseases and conditions in retinal photographs using deep neural networks. Nat Commun. 2021;12(1):1–13. https://www.nature.com/articles/s41467-021-25138-w. 10.1038/s41467-021-25138-w PMC8355164 34376678

[4] Wang J, Ji J, Zhang M, Lin J-W, Zhang G, Gong W, et al. Automated explainable multidimensional deep learning platform of retinal images for retinopathy of prematurity screening. JAMA Netw Open. 2021;4(5):1–12. https://jamanetwork.com/journals/jamanetworkopen/fullarticle/2779454. 10.1001/jamanetworkopen.2021.8758 PMC8100867 33950206

[5] Tang Y-W, Ji J, Lin J-W, Wang J, Wang Y, Liu Z, et al. Automatic detection of peripheral retinal lesions from ultrawide-field fundus images using deep learning. Asia Pac J Ophthalmol. 2023;12(3):284–92. https://journals.lww.com/apjoo/fulltext/2023/05000/automatic_detection_of_peripheral_retinal_lesions.4.aspx. 10.1097/APO.0000000000000599 36912572

[6] Liu G, Ding Q, Luo H, Sha M, Li X, Ju M (2022). Cx22: A new publicly available dataset for deep learning-based segmentation of cervical cytology images. Comput Biol Med.

[7] Zhou Y, Chen H, Xu J, Dou Q, Heng P-A, editors. IRNet: Instance Relation Network for Overlapping Cervical Cell Segmentation. Medical Image Computing and Computer Assisted Intervention – MICCAI 2019; 2019 2019//; Cham: Springer International Publishing.

[8] Liu Y, Zhang P, Song Q, Li A, Zhang P, Gui Z (2018). Automatic segmentation of cervical nuclei based on deep learning and a conditional random field. IEEE Access.

[9] Lu Z, Carneiro G, Bradley AP (2015). An improved joint optimization of multiple level set functions for the segmentation of overlapping cervical cells. IEEE Trans Image Process.

[10] Lu Z, Carneiro G, Bradley AP, Ushizima D, Nosrati MS, Bianchi AGC (2017). Evaluation of three algorithms for the segmentation of overlapping cervical cells. IEEE J Biomed Health Inform.

[11] Plissiti ME, Dimitrakopoulos P, Sfikas G, Nikou C, Krikoni O, Charchanti A, editors. Sipakmed: a new dataset for feature and image based classification of normal and pathological cervical cells in Pap Smear Images. 2018 25th IEEE International Conference on Image Processing (ICIP); 2018 7–10 Oct. 2018.

[12] Rezende MT, Silva R, Bernardo FdO, Tobias AHG, Oliveira PHC, Machado TM, et al. Cric searchable image database as a public platform for conventional pap smear cytology data. Sci Data. 2021;8(1):151. 10.1038/s41597-021-00933-8 PMC8192784 34112812

[13] Rahaman MM, Li C, Yao Y, Kulwa F, Wu X, Li X (2021). DeepCervix: a deep learning-based framework for the classification of cervical cells using hybrid deep feature fusion techniques. Comput Biol Med.

[14] Bhatt AR, Ganatra A, Kotecha K (2021). Cervical cancer detection in pap smear whole slide images using convNet with transfer learning and progressive resizing. PeerJ Computer Science.

[15] Nayar R, Wilbur DC (2015). The Pap test and Bethesda 2014. Acta Cytol.

[16] Zhang J, Liu Z, Du B, He J, Li G, Chen D (2019). Binary tree-like network with two-path Fusion Attention Feature for cervical cell nucleus segmentation. Comput Biol Med.

[17] Ronneberger O, Fischer P, Brox T. U-Net: Convolutional Networks for Biomedical Image Segmentation. ArXiv e-prints [Internet]. 2015; 1505. Available from: http://adsabs.harvard.edu/abs/2015arXiv150504597R. Accessed 1 Apr 2023.

[18] Zhou Z, Mahfuzur Rahman Siddiquee M, Tajbakhsh N, Liang J. UNet++: A Nested U-Net Architecture for Medical Image Segmentation. ArXiv e-prints [Internet]. 2018; 1807. Available from: http://adsabs.harvard.edu/abs/2018arXiv180710165Z. Accessed 15 Apr 2023. 10.1007/978-3-030-00889-5_1 PMC7329239 32613207

[19] Huang H, Lin L, Tong R, Hu H, Zhang Q, Iwamoto Y, et al. UNet 3+: A Full-Scale Connected UNet for Medical Image Segmentation. 2020:[arXiv:2004.08790 p.]. Available from: https://ui.adsabs.harvard.edu/abs/2020arXiv200408790H. Accessed 15 Apr 2023.

[20] Zhou Z-H, Li SZ, Jain AK (2015). Ensemble Learning. Encyclopedia of Biometrics.

[21] Chen L-C, Papandreou G, Schroff F, Adam H. Rethinking atrous convolution for semantic image segmentation. ArXiv e-prints [Internet]. 2017; 1706. Available from: http://adsabs.harvard.edu/abs/2017arXiv170605587C. Accessed 1 Apr 2023.

[22] Chen L-C, Zhu Y, Papandreou G, Schroff F, Adam H. Encoder-Decoder with atrous separable convolution for semantic image segmentation. arXiv e-prints [Internet]. 2018. Available from: https://ui.adsabs.harvard.edu/#abs/2018arXiv180202611C. Accessed 1 Apr 2023.

[23] Chen J, Lu Y, Yu Q, Luo X, Adeli E, Wang Y, et al. TransUNet: transformers make strong encoders for medical image segmentation. 2021 :[arXiv:2102.04306 p.]. Available from: https://ui.adsabs.harvard.edu/abs/2021arXiv210204306C. Accessed 1 Apr 2023.

[24] Xie E, Wang W, Yu Z, Anandkumar A, Alvarez JM, Luo P. SegFormer: Simple and efficient design for semantic segmentation with transformers. 2021:[arXiv:2105.15203 p.]. Available from: https://ui.adsabs.harvard.edu/abs/2021arXiv210515203X. Accessed 15 Apr 2023.

[25] Oktay O, Schlemper J, Le Folgoc L, Lee M, Heinrich M, Misawa K, et al. Attention U-Net: learning where to look for the pancreas. ArXiv e-prints [Internet]. 2018. Available from: https://ui.adsabs.harvard.edu/#abs/2018arXiv180403999O. Accessed 1 Apr 2023.

[26] Zahangir Alom M, Hasan M, Yakopcic C, Taha TM, Asari VK. Recurrent residual convolutional neural network based on U-Net (R2U-Net) for medical image segmentation. ArXiv e-prints [Internet]. 2018; 1802. Available from: http://adsabs.harvard.edu/abs/2018arXiv180206955Z. Accessed 1 Apr 2023.

[27] Transformer S. Swin-Transformer-Semantic-Segmentation 2020 [Available from: https://github.com/SwinTransformer/Swin-Transformer-Semantic-Segmentation. Accessed 10 Oct 2022.

[28] Sagi O, Rokach L (2018). Ensemble learning: a survey. WIREs Data Min Knowl Discovery.

[29] Jadon S. A survey of loss functions for semantic segmentation. 2020:[arXiv:2006.14822 p.]. Available from: https://ui.adsabs.harvard.edu/abs/2020arXiv200614822J. Accessed 15 May 2021.

[30] Kingma DP, Ba J. Adam: A Method for Stochastic Optimization. arXiv e-prints. 2014:arXiv:1412.6980. Accessed 6 Nov 2022.

[31] Zhang MR, Lucas J, Hinton G, Ba J. Lookahead Optimizer: k steps forward, 1 step back. arXiv e-prints [Internet]. 2019. Available from: https://ui.adsabs.harvard.edu/abs/2019arXiv190708610Z. Accessed 1 Apr 2023.

[32] Micikevicius P, Narang S, Alben J, Diamos G, Elsen E, Garcia D, et al. Mixed Precision Training. arXiv e-prints. 2017:arXiv:1710.03740.

[33] Guo C, Pleiss G, Sun Y, Weinberger KQ. On calibration of modern neural networks. Proceedings of the 34th International Conference on Machine Learning - Volume 70; Sydney, NSW, Australia. 3305518: JMLR.org; 2017. p. 1321–30.

[34] Parikh R, Mathai A, Parikh S, Chandra Sekhar G, Thomas R (2008). Understanding and using sensitivity, specificity and predictive values. Indian J Ophthalmol.

