# Supplementary material for: Automated cervical cell segmentation using deep ensemble learning
Source: BMC Med Imaging. 2023 Sep 21;23:137. doi: 10.1186/s12880-023-01096-1 (PMC10514950; doi:10.1186/s12880-023-01096-1)
Supplement: Supplementary file 1 — Additional file 1: Figure S1 and Figure S2. [file 12880_2023_1096_MOESM1_ESM.docx]

**Supplement materials**

**
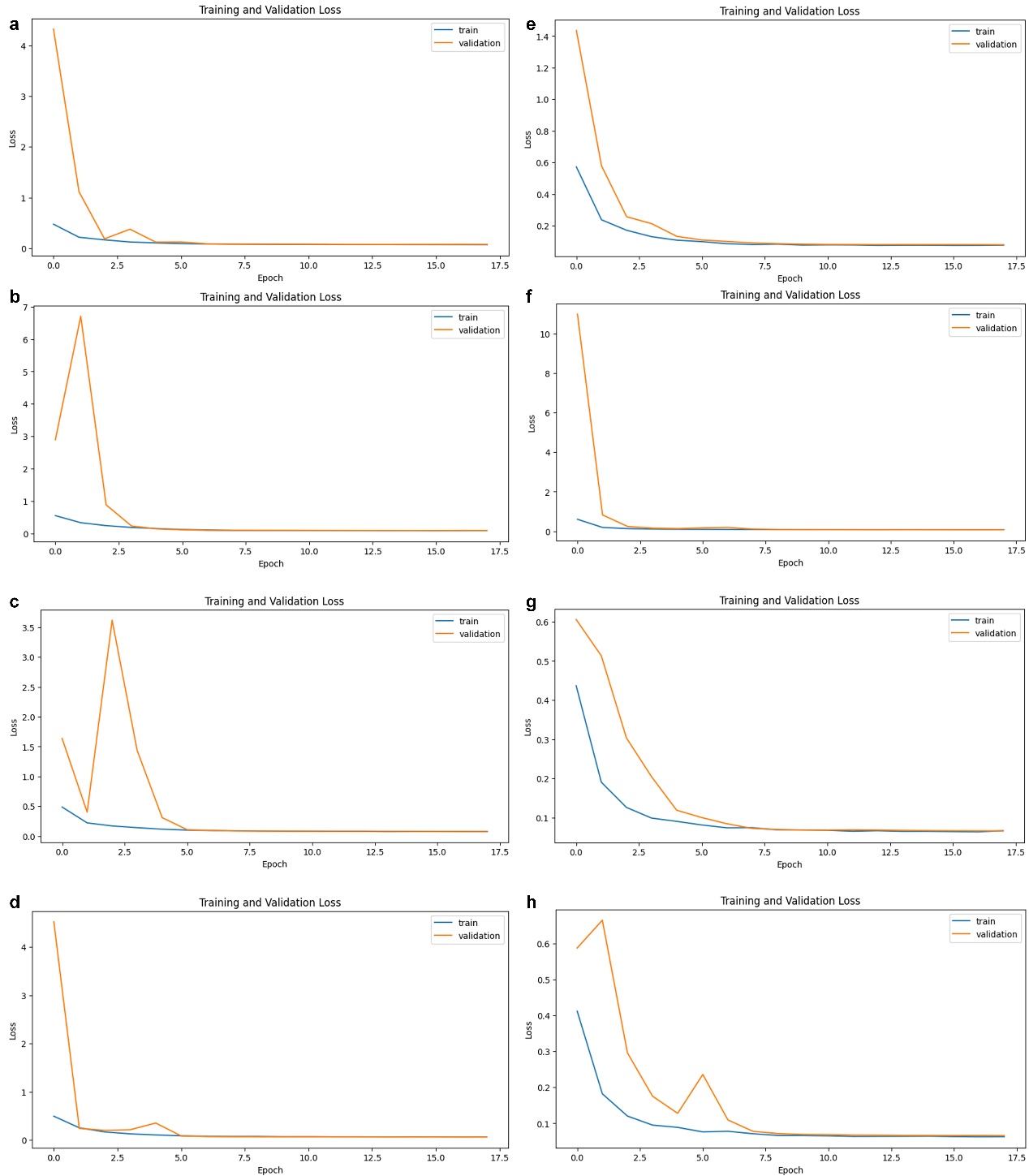
**

**Figure S1.** Training and validation losses of models of cytoplasm segmentation. (a) Unet_resnet34; (b) Unet_densenet121; (c) UnetPlusPlus_resnet34; (d) UnetPlusPlus_densenet121; (e) DeepLabV3_resnet34; (f) DeepLabV3_resnet50; (g) DeepLabV3Plus_resnet34; (h) DeepLabV3Plus_resnet50;

**
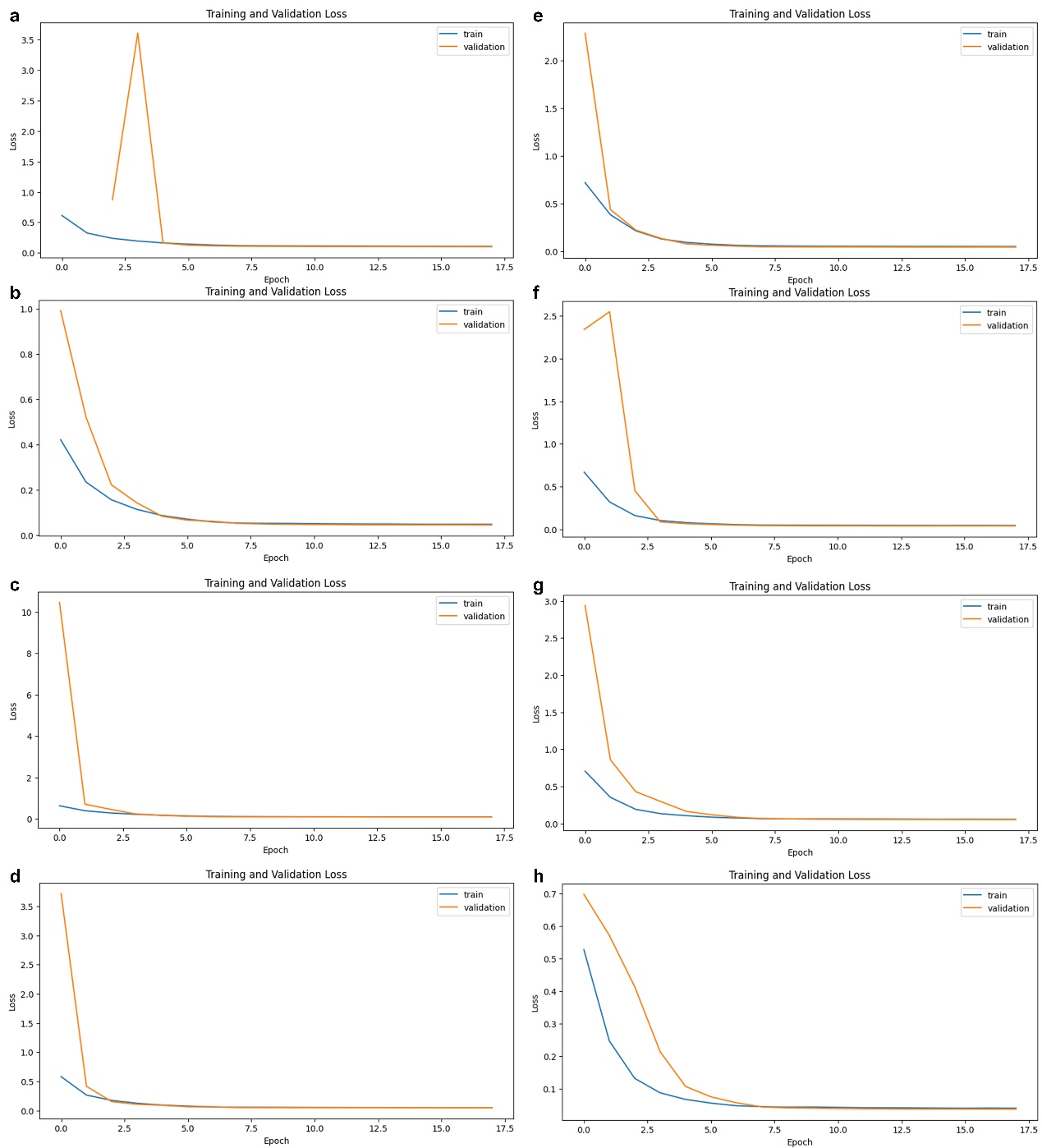
**

**Figure S2.** Training and validation losses of models of nucleus segmentation. (a) Unet_resnet34; (b) Unet_densenet121; (c) UnetPlusPlus_resnet34; (d) UnetPlusPlus_densenet121; (e) DeepLabV3_resnet34; (f) DeepLabV3_resnet50; (g) DeepLabV3Plus_resnet34; (h) DeepLabV3Plus_resnet50;
